# Association of Baseline Serum Soluble Tumour Necrosis Factor Receptor Levels with the Response of Rheumatoid Arthritis to Janus Kinase Inhibitor Therapy

**DOI:** 10.1155/2024/2898586

**Published:** 2024-04-27

**Authors:** Takahiro Yoshikawa, Tetsuya Furukawa, Teppei Hashimoto, Naoto Azuma, Kiyoshi Matsui

**Affiliations:** Department of Diabetes Endocrinology and Clinical Immunology, School of Medicine, Hyogo Medical University, 1-1, Mukogawa-cho, Nishinomiya, Hyogo 663-8501, Japan

## Abstract

**Aim:**

The aim of this study was to investigate whether cytokines associated with tumour necrosis factor- (TNF-) *α* and interleukin- (IL-) 6 signalling could predict rheumatoid arthritis (RA) clinical remission (CR) with Janus kinase inhibitor (JAKinib) treatment using the Simplified Disease Activity Index (SDAI).

**Methods:**

Eighty-nine patients with RA treated with JAKinibs were enrolled, and their clinical data were collected retrospectively. CR was defined as an SDAI ≤ 3.3 after 6 months of treatment with JAKinib. The serum samples of 89 patients were analysed for IL-6, soluble IL-6 receptor (sIL-6R), soluble gp130 (spg130), and soluble TNF receptor- (sTNFR-) I and sTNFR-II titres.

**Results:**

There were no significant differences in the baseline clinical parameters between the CR and non-CR groups. Serum levels of IL-6, sIL-6R, and sgp130 were not significantly different; whereas, the serum sTNFR-I and sTNFR-II levels were significantly lower in the CR group. Univariate and multivariate logistic regression analysis showed that the baseline log sTNFR II values (OR: 0.002; *p* = 0.034) were predictors of CR.

**Conclusions:**

Patients with RA can be stratified prior to JAKinib administration using serum sTNFR-I and sTNFR-II levels but not serum IL-6 axis cytokine levels (IL-6, sIL-6R, and sgp130).

## 1. Introduction

Rheumatoid arthritis (RA) is an autoimmune disease that causes persistent synovitis, which leads to joint destruction. Our understanding of its aetiology has advanced dramatically in recent decades [1], and combined histopathological and clinical observations suggest that there are different patterns of rheumatoid synovitis with tumour necrosis factor- (TNF-) *α* and interleukin-6 (IL-6) being the main players in the two most common types, myeloid and lymphoid synovitis, respectively [2, 3]. Thus, TNF-*α* and IL-6 are the two main cytokines thought to contribute most to the many pathogenic signalling pathways that cause RA and to determine the histological type of synovitis, and hence, response to treatment with IL-6, interferon (IFN), granulocyte-macrophage colony-stimulating factor (GM-CSF), and the common *γ*-chain cytokine family. Several cytokines involved in the pathogenesis of RA act via the Janus kinase (JAK) signalling transducer and transcription activator (STAT) pathway [4, 5]. The European League Against Rheumatism (EULAR) recommends that the treatment of RA, updated in 2019, include TNF-*α* and IL-6 inhibitors as biological disease-modifying antirheumatic drugs (bDMARDs) for patients with poor prognostic factors and insufficient control with conventional synthetic disease-modifying antirheumatic drugs (csDMARDs). Moreover, JAK inhibitors (JAKinibs) have also been recommended alongside biological agents [6]. However, to date, no biomarkers have been established for predicting the efficacy of JAKinib in RA. There is an urgent need to find sensitive biomarkers that predict the response of RA to JAKinib as this may help accelerate the next generation of RA treatment by helping optimize the distribution of therapeutic agents. Therefore, the aim of this study was to investigate whether cytokines associated with TNF-*α* and IL-6 signalling could predict RA clinical remission (CR) with JAKinib treatment using the Simplified Disease Activity Index (SDAI).

## 2. Materials and Methods

### 2.1. Patients

A total of 125 patients with RA who fulfilled the 2010 American College of Rheumatology (ACR)/EULAR classification criteria and were prescribed JAKinibs (tofacitinib and baricitinib) at our hospital from July 2013 to July 2021 were enrolled. The study period was 6 months after the initiation of JAKinib treatment. JAKinibs use was based on Japan College of Rheumatology guidelines and recommendations in use during the study period [7]. Thirty-six patients without a valid SDAI score at either baseline or follow-up (less than 6 months) and for whom serum tests were not available were excluded from the study. ACR criteria for CR based on the SDAI (SDAI < 3.3) were used to define remission. The study was conducted in accordance with the tenets of the Declaration of Helsinki and approved by the ethics committee of our institution. Informed consent was obtained from all patients in accordance with institutional policy (approval no. 3642; Hyogo Medical University).

### 2.2. Data Collection

In addition to the history of JAKinib use, we retrospectively reviewed cohort characteristics, including sex, age, disease duration, weekly dose of methotrexate (MTX), daily dose of prednisolone (PSL), and use of csDMARDs other than MTX at baseline. C-reactive protein (CRP) level and SDAI were also collected at baseline and after 6 months.

### 2.3. Measurement of Cytokines Associated with TNF-*α* and IL-6 Signalling

Baseline serum samples collected at the time of diagnosis were available for the 89 patients with RA. These samples were stored at −20°C until further analysis. Serum soluble TNF receptor- (sTNFR-) I (Human TNF RI/TNFRSF1A Quantikine ELISA Kit DRT100) and sTNFR-II (Human sTNF RII/TNFRSF1B Quantikine ELISA Kit DRT200) levels were measured as TNF-*α*-related cytokines and serum IL-6 (Human IL-6 Quantikine ELISA Kit, R&D systems), soluble IL-6 receptor (sIL-6R) (Human soluble IL-6R alpha Quantikine ELISA Kit, R&D systems), and soluble gp130 (sgp130) (Human soluble gp130 Quantikine ELISA Kit, R&D systems) levels were measured as cytokines associated with IL-6 signalling using specific ELISAs according to the manufacturer's instructions. sTNFR-I and sTNFR-II measurements have several advantages over the measurement of TNF-*α*. TNF-*α* is rapidly cleared from circulation and is often undetectable, whereas sTNFR-II is not. sTNFR-I and sTNFR-II are very stable in stored serum [8]. In addition, serum sTNFR-I and sTNFR-II levels correlate well with TNF-*α* levels [9, 10]. sTNFR levels in patients with RA are elevated compared to those in healthy individuals, patients with osteoarthritis, or even patients with other forms of inflammatory arthritis [11]. Moreover, the levels of sTNFR have been found to correlate with the severity of disease activity in patients with RA [12]. Therefore, serum sTNFR-I and sTNFR-II levels were measured instead of TNF-*α* levels.

### 2.4. Statistical Analyses

All variables are expressed as percentages for categorical data and as medians and interquartile ranges (IQRs) for nonnormal continuous data. Differences between groups were tested using the Mann–Whitney *U* test, depending on the distribution. Fisher's exact test was used to compare categorical data. Spearman's correlation analysis was used to assess the association between inflammatory biomarkers. Univariate logistic regression analysis was used to examine the association between the baseline variables and CR at 6 months. In this analysis, serum IL-6, sIL-6, sgp130, sTNFR-I, and sTNFR-II values were log-transformed to base 10 to correct for any bias.

Factors with at least borderline significance (*p* < 0.15) in the univariate analysis were included in the multivariate logistic regression analysis. All statistical analyses were performed using EZR 1.61 (Saitama Medical Center, Jichi Medical University, Saitama, Japan) [13], and *p* values of < 0.05 were considered statistically significant.

## 3. Results

### 3.1. Patient Background

The baseline patient characteristics are presented in Table 1. The median patient age was 62 years (IQR: 51–72), and the median disease duration was 6 years (IQR: 2–16). A total of 74 (83.1%) patients were female. The median SDAI was 18.9 (IQR: 12.7–27.9). The frequency of concomitant MTX use was 61 (68.5%), with a median dose of 10.0 (8.0–10.0) mg/week. The frequency of concomitant PSL use was 50 (56.2%), with a median dose of 5.0 (3.0–7.5) mg/day. Salazosulfapyridine (SASP) was used concomitantly in 32.6%, iguratimod (IGU) in 9.0%, bucillamine (BUC) in 9.0%, and tacrolimus (TAC) in 14.6% patients. The frequency of naïve use of bDMARDs and JAKinibs was 32 (36.0%). The proportion of biologic or JAKinib use before JAKinib was 39.3% for TNF inhibitor (TNFi), 25.0% for IL-6R inhibitor (IL-6Ri), 21.4% for abatacept, and 14.3% for another JAKinib (Table 1).

### 3.2. Outcome

SDAI CR was achieved in 21 (23.6%) patients 6 months after starting JAKinib treatment. Comparing the SDAI CR group with the non-CR group, MTX doses were significantly higher in the CR group than in the non-CR group. There were no significant differences in other baseline clinical parameters. PSL was discontinued within 6 months in 14.0% of all patients. There were no significant differences between the two groups. csDMARDs, including MTX, were continued throughout the study (Table 1).

### 3.3. Cytokines Associated with TNF-*α* and IL-6 Signalling

**Figure 1:**
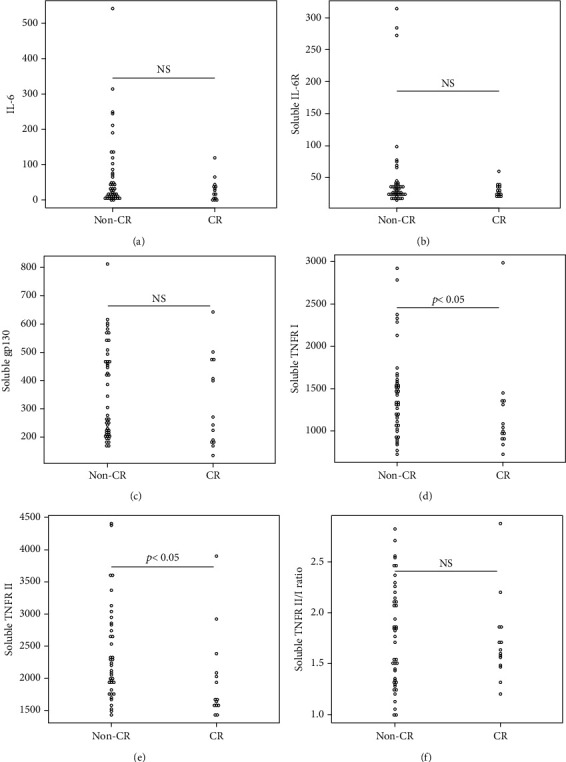
Comparison of various cytokines in the CR and non-CR groups. (a) interleukin-6 (IL-6), (b) soluble IL-6 receptor (IL-6R), (c) soluble gp130, (d) soluble tissue necrosis factor receptor-I (TNFR-I), (e) soluble TNFR-II and (f) soluble TNFR II/I ratio.

Overall, the median serum sTNFR-I level was 1314.0 (983.1–1628.2) pg/mL, and the median serum sTNFR-II level was 2194.4 (1723.8–3078.6) pg/mL. The CR group had significantly lower serum sTNFR-I and sTNFR-II levels than did the non-CR group (sTNFR-I: 1018.5 (918.7–1343.7) pg/mL vs. 1447.3 (1083.6–1635.2) pg/mL, *p* < 0.03; sTNFR-II; 1670.7 (1578.2–2056.9) pg/mL vs. 2312.8 (1936.5–3301.9) pg/mL, *p* < 0.005). In all the groups, the median serum IL-6, sIL-6R, and sgp130 levels at baseline were 27.7 (9.2–65.5) pg/mL, 30.6 (23.9–37.5) mg/mL, and 306.7 (214.6–471.3) ng/mL, respectively. In all the groups, the median serum IL-6, sIL-6R, and sgp130 levels at baseline were 27.7 (9.2–65.5) pg/mL, 30.6 (23.9–37.5) mg/mL, and 306.7 (214.6–471.3) ng/mL, respectively. However, the levels of these three cytokines were not significantly different between the two groups (Figure 1).

### 3.4. Factors Associated with CR at 6 Months after Starting JAKinibs

Univariate logistic regression analysis showed that achieving CR after 6 months of JAKinib treatment was significantly associated with baseline serum log sTNFR-II level (*p* = 0.034). Although not significant, lower baseline log sTNFR-I tended to be associated with a higher likelihood of achieving CR. In contrast, the other clinical parameters and cytokines were not associated with CR. We performed a multivariate analysis of the factors with at least borderline significance (*p* < 0.15) in the univariate analysis. The multivariate analysis identified baseline log sTNFR II values as predictors of CR (Table 2).

Serum sTNFR-I was weakly correlated with CRP, a marker of disease activity in RA, but not with the SDAI. Serum sTNFR-II levels did not correlate with CRP or SDAI. For cytokines associated with IL-6 signalling, sgp130 did not correlate with CRP and SDAI, while serum IL-6 and sIL-6R correlated with CRP but not with SDAI (Table 3).

## 4. Discussion

In this study, serum sTNFR-I and sTNFR-II levels as TNF-related cytokines and serum IL-6, sIL-6R, and sgp130 levels as IL-6 signalling-related cytokines were measured in patients with RA treated with JAKinibs. The results showed that TNF-related cytokines, such as serum sTNFR-I and sTNFR-II, were significantly lower in patients who achieved CR of RA based on SDAI after 6 months of treatment with JAKinibs. Moreover, multivariate logistic regression analysis showed that baseline log sTNFR II values (OR: 0.002; *p* = 0.034) were predictors of CR. However, IL-6 signalling-related cytokines did not differ significantly between the CR and non-CR groups. No clinical parameter at baseline was associated with CR. Moreover, there was no correlation between the sTNFR I/II levels and SDAI in RA in this study. TNF-*α* and IL-6 are the two main cytokines thought to be involved in many pathogenic signalling pathways that lead to RA [2, 3]. Rheumatologists consider TNF-*α* and IL-6 to be the main pathogenic targets for first-line treatment of patients with RA with DMARD-IR. They target TNF either directly or indirectly by inhibiting T-cell activation, adding IL-6 directly, or by downregulating the IFN pathway. Currently, treatment is dictated by comorbidities and patient preferences, as biomarkers have not been validated [14]. JAKinib was the first targeted synthetic disease-modifying antirheumatic drug for RA [15]. JAKinib inhibits the intracellular JAK/STAT pathway, which is involved in the pathogenesis and progression of RA [16]. Baricitinib and tofacitinib were the first JAKinibs recommended for moderate-to-severe RA after the failure of csDMARD therapy [6]. Several cytokines involved in the pathogenesis of RA, such as IL-6, IFN, GM-CSF, and the common *γ*-chain cytokine family, act through the JAK-STAT pathway [4, 5, 17]. Further, Valli et al. reported that IL-6 levels have been shown to be associated with response to tofacitinib treatment in RA [18]. Therefore, we initially predicted that IL-6 signalling-related cytokines would be relevant in predicting the therapeutic effect of JAKinib. However, none of the IL-6 signalling-related cytokines were associated with CR. In contrast, TNF-*α*-related cytokines have been found to be associated with the therapeutic effect of JAKinib, although TNF-*α* is involved in a signalling pathway independent of JAK. However, Valli et al. evaluated the efficacy of tofacitinib in RA using Disease Activity Score (DAS) 28-ESR and found that IL-6 and DAS28-ESR were significantly correlated. In the present study, we used SDAI to assess the efficacy of JAKinib; cytokines associated with IL-6 signalling, including IL-6, were not significantly correlated with SDAI. Although JAKinibs may indeed suppress IL-6 signalling, it is possible that IL-6 signalling-related cytokines are not predictive of JAKinib efficacy when SDAI is used as an efficacy indicator. TNF-*α* stimulates macrophages, T-cells, fibroblasts, and endothelial cells, leading to the release of other inflammatory cytokines, leukocyte migration into the synovium, and neovascularisation. IL-6, on the other hand, has been shown to cause B-cell proliferation and antibody production, induce T-cell differentiation into IL-17-secreting T-helper cells, and reduce regulatory T-cell differentiation. IL-6 also promotes angiogenesis and osteoclastogenesis. Recent integrative histopathological and clinical observations support the notion that rheumatoid synovitis has different patterns and that TNF-*α* and IL-6 are the primary causes of the two most common types of synovitis in RA, myeloid and lymphoid synovitis [2, 3]. TNF-*α* levels were found to correlate well with serum sTNFR-I and sTNFR-II levels in patients with RA [8–12]. Therefore, high serum sTNFR-I or sTNFR-II levels may indicate that a higher proportion of the synovium in patients with RA is myeloid. Dennis et al. found that an elevated myeloid axis in synovial tissue was significantly associated with a favourable clinical outcome with anti-TNF-*α* therapy, whereas an elevated lymphoid axis in synovial tissue was associated with a good response to anti-IL-6 therapy [2, 3]. Furthermore, the IL-6/IL-6R pathway signals via the JAK/STAT pathway, unlike the canonical nuclear factor kappa B pathway, which is primarily activated by TNF-*α* [19]. Although not directly proven, JAKinibs are expected to respond similarly to anti-IL-6 therapy in lymphoid synovitis. We believe that the effect of JAKinibs may be better in lymphoid-type synovitis but worse in myeloid-type synovitis and that patients with high serum sTNFR-I or sTNFR-II levels are less likely to achieve CR with JAKinibs based on this study.

This study had several limitations. First, the study was conducted retrospectively. However, the clinical and demographic characteristics at baseline were not significantly different between the CR and non-CR groups. A second limitation is that low serum sTNFR-I and sTNFR-II levels do not necessarily indicate low TNF-*α* gene expression. However, if there was no correlation between serum sTNFR I/II levels and TNF-*α* gene expression, there would be no significant difference between the CR and non-CR groups; therefore, serum sTNFR I/II levels and TNF-*α* gene expression may be correlated.

The identification of pretreatment predictors of response to JAKinibs, such as a combination of serum sTNFR-I and sTNFR-II levels, could easily be used in a tailored stratification approach, which is important in RA. Further studies with larger prospective cohorts are needed to increase the predictive power of our approach for clinical purposes.

## 5. Conclusions

Patients with RA can be stratified prior to JAKinib treatment using serum sTNFR-I and sTNFR-II levels, but not IL-6 axis cytokines (IL-6, sIL-6R, and sgp130).

## Figures and Tables

**Table 1:** Baseline characteristics of patients with rheumatoid arthritis.

		All	CR 24 W	Non-CR 24 W	*p* value
		*n* = 89	*n* = 21	*n* = 68	
Age (years)	Median [IQR]	62 [51–72]	64.0 [43.0–69.0]	62 [51.8–72.3]	0.392
Female (%) (*n*)		83.1 (74)	81.0 (17)	83.8 (57)	0.746
BMI	Median [IQR]	21.3 [18.6–24.3]	21.0 [18.7–23.0]	21.5 [18.6–24.4]	0.800
Duration (years)	Median [IQR]	6.0 [2.0–16.0]	4.0 [2.0–9.0]	8.0 [2.0–18.3]	0.178
Stage I/II/III/IV		18/20/11/21	6/6/2/2	12/14/9/19	0.270
Biologic- and JAKinib-naïve status (%) (*n*)		36.0 (32)	47.6 (10)	32.4 (22)	0.298
Biologic use history (%) (*n*)		59.6 (53)	47.6 (10)	63.2 (43)	0.216
JAKinib use history (%) (*n*)		9.0 (8)	4.8 (1)	10.3 (7)	0.675
Biologic and JAKinib use prior to JAKinib					
TNFi (%) (*n*)		39.3 (22)	63.6 (7)	32.6 (15)	0.397
IL-6Ri (%) (*n*)		25.0 (14)	18.2 (2)	26.1 (12)	
Abatacept (%) (*n*)		21.4 (12)	9.1 (1)	23.9 (11)	
Another JAKinib (%) (*n*)		14.3 (8)	9.1 (1)	15.2 (7)	
JAKinib					
Tofacitinib (%) (*n*)		37.1 (33)	47.6 (10)	33.8 (23)	0.305
Baricitinib (%) (*n*)		62.9 (55)	52.4 (11)	66.2 (45)	
MTX use (%) (*n*)		68.5 (61)	76.2 (16)	66.2 (45)	0.435
MTX dose (mg/week)	Median [IQR]	10.0 [8.0–10.0]	10.0 [10.0–12.0]	8.0 [8.0–10.0]	0.0469
PSL use (%) (*n*)		56.2 (50)	42.9 (9)	60.3 (41)	0.206
PSL dose (mg/day)	Median [IQR]	5.0 [3.0–7.5]	5.0 [5.0–7.5]	5.0 [3.0–7.5]	0.487
PSL discontinuation within 6 months (%) (*n*)		14.0 (7)	22.2 (2)	12.2 (5)	0.595
SASP use (%)		32.6 (29)	28.6 (6)	33.8 (23)	0.792
IGU use (%)		9.0 (10)	4.8 (1)	13.2 (9)	0.441
BUC use (%)		9.0 (8)	4.8 (1)	10.3 (7)	0.675
TAC use (%)		14.6 (13)	4.8 (1)	17.6 (12)	0.386
ESR (mm/h)	Median [IQR]	35.0 [10.8–67.0]	33.5 [13.8–71.0]	35.0 [9.8–65.5]	0.487
CRP (mg/mL)	Median [IQR]	1.46 [0.24–3.12]	2.00 [0.82–2.60]	1.11 [0.15–3.13]	0.276
SDAI	Median [IQR]	18.9 [12.7–27.9]	19.0 [13.7–23.9]	18.5 [12.7–30.5]	0.850
eGFR-Crea (mL/min/1.73 m^2^)	Median [IQR]	84.5 [70.0–104.5]	101.5 [76.5–116.0]	82.0 [68.5–95.3]	0.0625

Abbreviations: CR: clinical remission; BMI: body mass index; IQR: interquartile range; SDAI: Simplified Disease Activity Index; eGFR-Crea: estimated glomerular filtration rate-creatinine; CRP: C-reactive protein; ESR: erythrocyte sedimentation rate; JAKi naïve: Janus kinase inhibitor naïve; JAKinib: Janus kinase inhibitor; MTX: methotrexate; PSL: prednisolone; SASP: salazosulfapyridine; IGU: iguratimod; BUC: bucillamine; TAC: tacrolimus; TNFi: tumour necrosing factor inhibitor; IL-6Ri: interleukin-6 receptor inhibitor.

**Table 2:** Univariate and multivariate logistic regression analysis for outcomes.

	OR	95% CI	*p* value
Univariate analysis			
Age (years)	0.973	[0.942–1.010]	0.104
Female (%)	0.820	[0.231–2.910]	0.759
BMI	0.968	[0.847–1.110]	0.627
Duration (year)	0.952	[0.897–1.010]	0.110
Stage		I reference	
II	0.857	[0.218–3.370]	0.825
III	0.444	[0.072–2.740]	0.382
IV	0.211	[0.036–1.220]	0.082
CRP (mg/dL)	1.070	[0.888–1.290]	0.475
SDAI	0.985	[0.942–1.030]	0.491
Biologic- and JAKinib-naïve status	1.900	[0.702–5.150]	0.206
JAKinib	Baricitinib	Reference	
Tofacitinib	1.780	[0.659–4.800]	0.256
MTX use	1.640	[0.532–5.300]	0.390
PSL use	0.476	[0.176–1.290]	0.143
SASP use	0.783	[0.268-2.290]	0.654
IGU use	0.328	[0.039-2.750]	0.304
BUC use	0.436	[0.051-3.760]	0.450
TAC use	0.233	[0.029–1.910]	0.175
Log IL-6 at baseline	0.504	[0.187–1.360]	0.176
Log sIL-6R at baseline	0.213	[0.011–4.000]	0.301
Log sgp130 at baseline	0.237	[0.011–5.340]	0.365
sTNFR II/I ratio at baseline	0.808	[0.222–2.940]	0.746
Log sTNFR II at baseline	0.002	[0.0000653–0.634]	0.034
Log sTNFR I at baseline	0.013	[0.000126–1.300]	0.065
Multivariate analysis			
Log sTNFR II at baseline	0.001	[0.000000251–0.458]	0.027

Abbreviations: OR: odds ratio; CI: confidence interval; BMI: body mass index; SDAI: Simplified Disease Activity Index; CRP: C-reactive protein; JAKinib: Janus kinase inhibitor; MTX: methotrexate; PSL: prednisolone; IL-6: interleukin-6; sIL-6R: soluble IL-6 receptor; sgp130: soluble gp130; sTNFR-I: soluble tissue necrosis factor receptor-I; sTNFR-II: soluble tissue necrosis factor receptor-II; SASP: salazosulfapyridine; IGU: iguratimod; BUC: bucillamine; TAC: tacrolimus.

**Table 3:** Correlation between sTNFR levels and markers of disease activity.

*r* [*p* value]	CRP	SDAI
Log sTNFR I	0.344 [<0.008]	0.174 [0.200]
Log sTNFR II	0.066 [0.616]	0.186 [0.171]
Log IL-6	0.395 [<0.002]	0.139 [0.307]
Log sIL-6R	-0.254 [<0.05]	-0.182 [0.181]
Log sgp130	0.033 [0.800]	-0.084 [0.541]

Abbreviations: SDAI: Simplified Disease Activity Index; CRP: C-reactive protein; sTNFR-I: soluble tissue necrosis factor receptor-I; sTNFR-II: soluble tissue necrosis factor receptor-II; IL-6: interleukin-6; sIL-6R: soluble IL-6 receptor; sgp130: soluble gp130.

## Data Availability

The datasets used and/or analysed during the current study are available from the corresponding author upon reasonable request.
